# Current experiences and educational preferences of general practitioners and staff caring for people with dementia living in residential facilities

**DOI:** 10.1186/1471-2318-9-36

**Published:** 2009-08-12

**Authors:** Christopher Beer, Barbara Horner, Osvaldo P Almeida, Samuel Scherer, Nicola T Lautenschlager, Nick Bretland, Penelope Flett, Frank Schaper, Leon Flicker

**Affiliations:** 1 Western Australian Centre for Health & Ageing, Western Australian Institute for Medical Research, Perth, Australia; 2 Centre for Medical Research, University of Western Australia, Perth, Western Australia, Australia; 3 School of Medicine and Pharmacology, University of Western Australia, Perth, Western Australia, Australia; 4 Centre for Research on Ageing, Curtin University of Technology, Perth, Western Australia, Australia; 5 School of Psychiatry and Neurosciences, University of Western Australia, Perth, Western Australia, Australia; 6 Royal Freemasons Homes of Victoria, Melbourne, Victoria, Australia; 7 Academic Unit for Psychiatry of Old Age, St Vincent's Health, Department of Psychiatry, University of Melbourne, Victoria, Australia; 8 Rowethorpe Medical Centre, Bentley, Western Australia, Australia; 9 Brightwater Care Group (Inc), Perth, Western Australian, Australia; 10 Alzheimer's Australia WA Ltd, Perth, Western Australia, Australia

## Abstract

**Background:**

Residential care is important for older adults, particularly for those with advanced dementia and their families. Education interventions that achieve sustainable improvement in the care of older adults are critical to quality care. There are few systematic data available regarding the educational needs of Residential Care Facility (RCF) staff and General Practitioners (GPs) relating to dementia, or the sustainability of educational interventions. We sought to determine participation in dementia education, perceived levels of current knowledge regarding dementia, perceived unmet educational needs, current barriers, facilitators and preferences for dementia education.

**Methods:**

A mixed methods study design was utilised. A survey was distributed to a convenience sample of general practitioners, and staff in 223 consecutive residential care facilities in Perth, Western Australia. Responses were received from 102 RCF staff working in 10 facilities (out of 33 facilities who agreed to distribute the survey) and 202 GPs (19% of metropolitan GPs). Quantitative survey data were summarised descriptively and chi squared statistics were used to analyse the distribution of categorical variables. Qualitative data were collected from general practitioners, staff in residential care facilities and family carers of people with dementia utilizing individual interviews, surveys and focus groups. Qualitative data were analysed thematically.

**Results:**

Among RCF staff and GPs attending RCF, participation in dementia education was high, and knowledge levels generally perceived as good. The individual experiences and needs of people with dementia and their families were emphasised. Participants identified the need for a person centred philosophy to underpin educational interventions. Limited time was a frequently mentioned barrier, especially in relation to attending dementia care education. Perceived educational needs relating to behaviours of concern, communication, knowledge regarding dementia, aspects of person centred care, system factors and the multidisciplinary team were consistently and frequently cited. Small group education which is flexible, individualized, practical and case based was sought.

**Conclusion:**

The effectiveness and sustainability of an educational intervention based on these findings needs to be tested. In addition, future interventions should focus on supporting cultural change to facilitate sustainable improvements in care.

## Background

Dementia is estimated to affect 0.9% of Australians and is now the leading cause of non-fatal disease burden among older Australians. [1,2] Prevalence is strongly age-related, with estimated prevalence rates of 6.5% in people aged 65 years and over, and 22.4% in people aged 85 years and over. [2] Australia has a complex system of community care provision, reflecting the preference of older people to remain in their own homes. [3] However, although most people with dementia are living in households (57%), many live in residential care facilities (43%). [2] Conversely, a large proportion of RCF residents have dementia (48%), and in fact this is the commonest medical problem affecting older people in residential care. [2,4] Ninety-six per cent of people with dementia living in care accommodation in Australia have moderate or severe dementia, compared to only 7% of people with dementia living in households. [2] Unmet needs appear to be common among residents with dementia living in care facilities. [5] Informed residential care is thus important for older adults with dementia, particularly for those with advanced dementia, and their families.

Dementia is the most common problem managed by GPs at RCF consultations. [6] The ability of RCF staff and GPs to identify and respond to dementia is of critical importance. Training programs in dementia care for RCF staff are common and have been systematically reviewed. [7] However the evidence identified was mostly published in the United States, and frequent methodological weaknesses limited the conclusions which could be drawn.

Several recent initiatives have aimed to improve quality of care and facilitate access to GP services by residents in Australian RCF (including the Aged Care GP Panels Initiative, the Enhanced Primary Care program, and an expanded role of palliative care). However many GPs still find providing RCF services unattractive. [8]

GPs in Australia are required to undertake Continuing Professional Development (CPD). To be eligible for CPD credit, activities must be prospectively approved, providing evidence of need, proposed objectives and evaluation, and reinforcing activities. Previous survey data showed that GPs have reasonable knowledge regarding dementia but that there were knowledge gaps regarding diagnosis and management of dementia. [9] In addition to educational programs, assessment protocols were requested. Interestingly GPs with better knowledge, and those with a greater proportion of consultations with older people, were more likely to seek further education. Therapeutic nihilism, desire to avoid risk, resourcing and competence have been identified as barriers to GPs participating in shared care models in the UK, and may also be relevant in Australia. [10]

The need to improve care of people with dementia living in residential facilities has been recognised and there has been a significant investment in dementia training in Australia through the 2005 Dementia Budget Initiative and other ongoing initiatives. However most of these initiatives have been aimed at specific groups of workers (such as direct care workers, or various health professionals) rather than multidisciplinary teams working in the same RCF. There are few systematic data regarding the content, sponsor, access, incentives to participation, known barriers and cost of currently available dementia education in Australia. In addition, there is also evidence that although there are many training programs currently offered these do not necessarily meet the perceived needs of learners. The National Stocktake [11] of currently available dementia education collected data from providers and stakeholders using surveys and focus groups. There was a lack of standardization between courses and problems were identified with both content and delivery of educational programs. Costs (especially for lower paid workers) and staff shortages were barriers to learning. In addition, the currently offered courses often used didactic and inflexible teaching. This is at odds with the general consensus regarding the value of interactive learning methodologies. [12]

In this study, we aimed to collect data to inform development of a sustainable educational intervention for RCF staff and GPs based on the perceived needs and preferences of learners. We sought to:

• determine current participation in dementia education and perceived levels of current knowledge regarding dementia,

• determine barriers to and facilitators of improved dementia care,

• determine perceived educational needs relating to residential care for people with dementia, and

• explore preferred methods of educational delivery.

## Methods

A mixed methods design, incorporating collection of both quantitative and qualitative data, was utilised.

### Quantitative Data Collection, Handling and Analysis

A survey was distributed to GPs and RCF staff in metropolitan Perth. Questions were framed in relation to the study aims using a tick box design to collect basic demographic data (gender, age range and whether English spoken as a first language). RCF staff were also asked to indicate their role (Direct care, Clinical care, Support services or Management), number of years worked in aged care, number of residents cared for, and number of residents cared for with diagnosed or suspected dementia. GPs were also asked to indicate their Division and suburb, and whether or not they attended patients with dementia who are living in residential care facilities, and if so, how many. Respondents were asked to rate their current knowledge of dementia ('not good', 'good', 'very good' or 'unsure') and to indicate whether they had participated in dementia education programs and how they rated those programs ('not good', 'good', 'very good' or 'unsure'). Participants were asked to indicate their preferred method of delivery for educational programs (Workshops, Internet website, Poster, Booklet, Other).

A convenience sample (reliant on distribution of the survey by third parties) was used. An attempt was made to contact all multiple site aged care providers (41 providers providing care in 184 facilities) and single-site facilities (39) in the Perth metropolitan area, requesting to speak with the Manager or Director of Nursing. Providers and facilities who agreed then distributed the survey to their staff. Thirteen multi-site aged care providers and 20 single site aged care facilities agreed to distribute the survey. The survey was also sent to the six Perth metropolitan Divisions of General Practice (Canning, Coastal, Fremantle, Osborne, Perth and Hills, Rockingham), who in turn distributed the survey to all GPs on their registers (around 1050 GPs). Four Divisions distributed the survey by post and 2 Divisions distributed the survey by facsimile. Surveys were distributed between September and November 2007. The closing date for return of surveys was set 3 weeks after distribution by each Division.

All survey responses, including incomplete surveys, were collated. Not all respondents answered all fields, hence the total number of responses for some survey fields varied. Descriptive statistics were used to summarize the quantitative survey results. Pearson chi-square statistics was used to analyze categorical variables.

### Qualitative Data Collection, Handling and Analysis

The surveys also utilised open ended questions to further explore current levels of knowledge, needs and preferences in relation to dementia education. Respondents were asked: 'In what areas do you think you need more knowledge in relation to working with people with dementia?'; 'What topics do you think should be included in an educational program for staff who are working with people with dementia?'; 'What will be the barriers to participation in an educational program?'; and 'Do you have any suggestions for the content of educational programs for GPs?'. The survey was written in English and translations to other languages were not provided. Surveys were anonymous, but provision was made for GPs to record their personal details if willing to be contacted regarding participation in individual interviews or focus groups.

An Expert Reference Group (ERG) was formed (see acknowledgments) comprising state and national experts in dementia care and dementia education. Terms of reference included identifying content and delivery methods for dementia education in residential care, and current barriers to education. Research staff kept a research journal recording any feedback received from Facility Managers regarding educational needs and preferences. This was collated and reflected upon during the study.

Individual interview and focus group guidelines were drafted and revised in light of the survey data and feedback from the ERG. Interviews and focus groups were conducted in care facilities, on university premises, or at participants' offices or homes. The interviews and focus groups aimed to determine perceived unmet educational needs and preferred content and delivery of education. GP participants were recruited from survey responses. Invitations to participate in individual interviews and/or focus groups for staff and carers of people with dementia were distributed by facility managers and Alzheimer's Australia WA. Carers of people with dementia were invited to share their own experiences, opinions and feelings (rather than acting as a proxy for the person with dementia). The interviews and focus group were facilitated by a social researcher with experience in mental health, training in conducting interviews and focus groups, and experience in qualitative research methodologies. The GP focus group was co-facilitated by a GP who has experience in dementia care.

Individual interviews, focus groups and meetings of the ERG were recorded and transcribed. Free text responses to the open ended survey questions, and notes made by study staff recording feedback from facility managers, were also transcribed.

Transcripts were coded line by line. Codes were assigned relating to thematic content, and a sub-code could be assigned when required. Initial coding of 13 transcripts was completed independently by the social researcher (who does not have specific experience in dementia care) and one of the authors (CB or BH, who are academic clinicians with experience in dementia care). This approach was prospectively chosen to minimise bias and support face validity of the qualitative analysis. Analysis continued until "saturation" (the qualitative researcher's impression that no new themes are emerging) occurred. A formal meeting was then held to review the initial individual analyses.

Consensus was reached regarding the emerging themes and sub-themes. The remaining data were independently coded by two people (the social researcher, and CB or BH). One coder then reviewed the two independent sets of codes, discussing any areas of uncertainty. Initial thematic analysis of final codes, by grouping of similar codes, was independently completed by two of the authors (BH, CB). A second formal meeting was then held to confirm consensus regarding the thematic analysis. A final thematic analysis was then drafted. Critical feedback was then sought from the remaining authors.

### Ethical considerations

The Human Research Ethics Committee at the University of Western Australia approved this study (RA 4/1/1685). Each participant in focus groups and individual interviews provided written informed consent.

## Results

### Quantitative Survey Results: Current Knowledge and Participation in Dementia Education

RCF staff returned 102 surveys. Ten RCFs returned an average of 10 surveys each. Determining the response rate is difficult for RCFs, as we relied on providers and facility managers to distribute the survey and did not confirm how many surveys were distributed. Responses were received from 30% of facilities who agreed to distribute the survey.

RCF staff respondents were most often female and in middle age groups. (Table 1) The majority (56%) worked in direct care or clinical care (24%) roles. The remainder worked in support services (15%), leadership and management (3%) or multiple (5%) roles. One in five of the respondents did not speak English as their first language. Although nearly half of respondents (46%) had less than five years experience, over one third of respondents (35%) were very experienced (having more than ten years experience). Twelve respondents (13% of those specifying gender) were male. Only five men answered the question regarding their first language, but 4 of those men said that English was not their first language. RCF respondents indicated that they cared for, on average, 52 residents. These data varied widely according to the facility type and the respondent's role. Respondents indicated that the majority of residents they cared for had either diagnosed or suspected dementia. On average, RCF Staff respondents reported that 70% of the residents they cared for had diagnosed or suspected dementia.

**Table 1:** Demographics of Survey Respondent, and Perceived Current Knowledge of Dementia and Preferences for Dementia Education

	**RCF staff** **n = 102** n (%*)	**GP** **n = 202** n (%*)
Gender Female	83 (87)	89 (45)

Aged		

25 years and under	5 (5)	0 (0)
26 to 35 years	10 (10)	18 (9)
36 to 45 years	37 (37)	63 (32)
46 to 55 years	26 (26)	65 (33)
56 to 65 years	22 (22)	43 (22)
66 years and over	1 (1)	11 (6)

English as first language	83 (82)	157 (81)

Self-rating of knowledge about dementia		

Very good	38 (38)	19 (10)
Good	49 (49)	112 (57)
Not good	11 (11)	49 (25)
Not sure	3 (3)	16 (8)

Have attended a dementia education program	80 (78)	86 (43)

Rated that program		

Very good	40 (51)	19 (23)
Good	36 (46)	57 (70)
Not good or not sure	2 (3)	6 (7)

Preferred delivery method		

Workshop	61 (62)	107 (57)
Internet website	2 (2)	13 (7)
Poster	0 (0)	0 (0)
Booklet	3 (3)	12 (6)
Workshop + Internet website, poster, or booklet	29 (30)	50 (27)
Other	3 (3)	6 (3)

* not all respondents answered all questions

Three quarters of RCF staff who responded had attended some training about dementia. (Table 1) Nearly all rated such educational programs as 'good' or 'very good'. Furthermore, most respondents rated their current knowledge about dementia as 'good'. RCF staff preferred education to be delivered as a workshop. About a third of respondents indicated a preference for multiple delivery methods, usually workshop and booklet. Visual learning materials for display in common areas ("posters") were chosen infrequently. Preference of RCF staff for electronic delivery (Internet website, or Internet and another methodology) was infrequent and similar in staff aged less than 46 (n = 5; 10%), and those aged 46 years and over (n = 4; 8%; Chi = 0.12, p = 0.73).

GPs from all Divisions responded to the survey, returning 202 surveys. Nineteen per cent of Perth GPs responded to the survey. Men and women were approximately evenly represented. (Table 1) One in five of the GPs who responded indicated that they did not speak English as their first language. Most GPs were older, and accordingly, most GPs who responded were very experienced (only 19% has less than 11 eleven years experience). More than half of the GP respondents (57%) did not currently care for people with dementia (PWD) living in residential care facilities. For those that did care for PWD living in RCF, the average case load was 21.

Just over half (57%) of the GPs who responded had not attended an educational program in dementia. (Table 1). While most (67%) said their level of knowledge regarding dementia was "very good" or "good", a surprisingly high percentage (25%) said that their knowledge was "not good", and a further 8% were uncertain. Workshops were the preferred method of delivery. Another popular option was workshop with booklet. Although a preference for Internet based delivery was more common than among RCF staff, the majority still preferred workshop based delivery. GPs aged 45 years and younger were more likely to indicate a preference for electronic delivery of education (Internet website, or Internet and another methodology, n = 26, 34%) compared to older GPs (n = 18, 17%, Chi = 7.25, p < 0.01). GPs who had not participated in an educational program were less likely to report "good" or very good' perceived knowledge (n = 58, 52%) compared to GPs who had participated in an educational program (n = 72, 86%, chi = 19.37, p < 0.001).

GPs who responded to the survey and were attending RCF tended to be older, and were more often male, than GPs not attending RCF (52% of male respondents reported attending RCF, compared with 32% of female respondents). Age specific proportions of GPs reporting caring for PWD in RCF were; 35 years and less 22%, 36–45 years 23%, 46–55 years 55% and 56 years and over 59%). GPs attending RCF were more likely to have attended an educational program and to perceive their knowledge as good or very good. (Table 2)

**Table 2:** Characteristics of GPs attending RCF compared with those not attending RCF

	Attends RCF n (%)	Does not attend RCF n (%)	Statistic	p
Male Gender	57 (66%)	51 (46%)	Chi = 8.443 Df = 1	0.004

Age				
<55	55 (63%)	91 (81%)	Chi = 7.47	0.006
56+	32 (37%)	22 (19%)	Df = 1	

English as first language	69 (82%)	88 (81%)	Chi = 0.062 Df = 1	0.803

Rate Knowledge				
VG or good	68 (80%)	63 (57%)	Chi = 11.73	<0.001
NG or unsure	17 (20%)	48 (43%)	Df = 1	

Have Participated in Dementia	53 (62%)	33 (29%)	Chi = 20.483	<0.001
Educational Program			Df = 1	

Chi = Pearson Chi squared; VG = 'very good'; NG = 'not good'

### Qualitative Results: Perceived Educational Needs, and Educational Preferences

In addition to open ended survey questions, qualitative data were available from individual interviews with 6 family carers, 5 GPs and 4 RCF staff; focus groups of 4 family carers, 9 GPs and 7 RCF staff, two meetings of the ERG and feedback recorded from 10 facility managers. There were 1829 individual codes. Most codes (780; 43%) were from GPs. Similar quantities of data were available from family carers (483 codes; 26%) and RCF staff (482 codes; 26%). Remaining data were from the ERG and feedback recorded from facility managers (84 codes; 5%).

Participants felt that education relating to dementia and dementia care should be underpinned by a clear guiding philosophy. In this respect participants asserted the importance of individualised, respectful person centred care as the foundation of educational interventions. A person centred approach was seen as facilitating high quality dementia care. Participants emphasised that a flexible approach, with a focus on understanding the personal history of the resident, facilitated care delivery. Understanding dementia as a "journey" was felt to facilitate individualised, person centred care and indicated the importance of changing needs.

*'I think it's a misnomer and I think it's a misleading issue too this whole thing of stages, it makes us think that there is, think in stages and there aren't, I mean people are just on a journey' *(ERG member)

Although negative aspects of care in residential facilities were recognised, including the indignities and costs of dementia care in personal terms, the potential for learning was emphasised.

*'It's taken you quite a while to learn what we learnt and we can still learn' *(Family Carer)

Participants identified multiple barriers to improving dementia care, and to participation in dementia education. System factors, including the complexity of aged care, and workforce factors, were frequently emphasised by participants. System factors ranged from local matters, such as appointment and review systems to more universal issues such as the available funding. Participants viewed a range of work force issues as potential barriers, including medical workforce shortages and rapid turnover of staff in residential care facilities.

*'But with the attrition of staff leaving the industry, so new people coming in and people moving on that you're constantly having this uphill battle of educating your staff' *(RCF Staff member)

Time constraints, and lack of communication were frequently mentioned as potential barriers, for example when lack of communication allows incorrect expectations to persist. Conflicting interests between workforce groups were also cited as barriers to best practice.

*'often the knowledge is there about which drugs you can use and how and you know to avoid too much medication but it's just that you either don't get told, or you can't do behavioural management – all they want is something to knock 'em out' *(GP)

Several potential barriers were also identified as facilitators. These included improved communication, and better organisation.

*'I think if you run your individual practice sensibly and you do regular comprehensive medical assessments then you can make it pay as well' *(GP)

Participants accepted that most people delivering care are usually well intentioned. However participants emphasized the importance of leadership in facilitating provision of quality care. Management leadership was cited as a potential facilitator of cultural change in residential care.

*'I think the biggest gap has come from the fact that quite often the care staff go and learn this information and they'll usually really enjoy it when they're at the course. When they come back into their environment, and it's set up in such a way that the culture's so hard to change because you almost need your higher management people to also understand that there needs to be the flexibility and all that kind of stuff as well' *(RCF Staff member)

Data from all sources provided evidence of a perceived need for improved knowledge of aspects of dementia and dementia care.

*'it's frustrating for me and it's frustrating for the residents if you don't have any knowledge' *(RCF Staff member)

In addition to knowledge based education regarding dementia itself, GPs and care staff identified education relating to other common health problems in people with dementia as being important. These included mental health issues, skin problems, pain and continence. Other important considerations included palliative approaches to care and the legal framework for care. There was recurrent emphasis on the need for education regarding assessment and care planning, both at the level of care staff, and professional staff. Behaviours of concern were consistently cited as an area of particular importance. Communication, similarly, was frequently identified as being of central importance. Communication issues related to inter-professional communication, communication with people with dementia, and also communication with family carers. Finally there were pragmatic requests for resources and education to assist people with dementia, their families and workers in negotiating various aspects of the aged care system.

There was some variation in preferred educational content for families, GPs and RCF staff. For example, RCF staff identified a need for education in strategies to engage people with dementia, and support for leadership in aged care facilities, more often than GPs. GPs cited issues relating to medication management and legal issues more frequently than RCF staff.

Participants emphasized the diversity of needs among both residential care staff and general practitioners, suggesting flexible, modular approaches to educational delivery. The need for individualized approaches related not only to prior learning, but also to individual preferences for educational delivery. Some participants suggested that external contributors were important in determining the curriculum for educational interventions. Participants, both GPs and RCF staff, generally favoured small group, interactive learning with a focus on opportunities for mentoring.

*'I think if you do give the people the opportunity to brainstorm solutions for their own problems they're usually very good. The care staff, particularly the ones who are really interested in finding answers to the problems themselves' *(RCF Staff member)

Case based learning was suggested for both face to face group learning, and for electronic delivery. Participants emphasised the need to ensure educational interventions are sustainable.

Unifying themes were found consistently and frequently in the qualitative data from each participant group, and across the survey, interview and focus group data. Behaviours of concern, communication, dementia knowledge and person-centred care were important to all groups of respondents. The importance of organisational factors such as support for effective multidisciplinary teamwork, communication and leadership were also consistently emphasized. However variability in experiences and needs across the sector were also emphasised, highlighting the importance of an individualized approach and identifying local barriers and solutions.

## Discussion

Study participants supported the goal of providing high quality care which focuses on the individual needs of people with dementia. In fact, several participants explicitly endorsed provision of care which is "person centred". [13] Thus the study findings accord with the currently available guidelines regarding care delivery in residential facilities. It is reassuring that not only is participation in educational opportunities high, but respondents working in and attending RCF generally perceived themselves as having good levels of knowledge regarding dementia. However, given the high prevalence of dementia in Australian RCF, the fact that 20% of RCF staff do not have specific dementia training is concerning. A quarter of GPs indicating that their knowledge is "not good" is also of concern. Perhaps some of these GPs care for small numbers of older people. However GPs caring for few older people are likely to become increasingly scarce given the ageing of our population. It will be difficult for those GPs to provide leadership in diagnosis and clinical care for PWD if their knowledge is limited. Other GPs may hold nihilistic attitudes.

The survey data tend to confirm anecdotal perceptions of increasing diversity in the aged care RCF workforce. The proportion of people reporting that English is not their first language (18%) appears higher than in the general Western Australian population (of which only 13% reported speaking a language other than English at home in the 2006 census). This heterogeneity may present challenges in the design of future educational interventions.

Findings of our study are consistent with the available evidence, which frequently identifies limited resources, and staff turnover as barriers to education in residential care. [12] Despite staff turnover being noted as a barrier in the qualitative responses, over one third of RCF staff who responded to the survey were very experienced (more than ten years) indicating a stable core of staff who may be targeted by educational interventions. Our results are also consistent with work showing that GPs commonly cite managing behaviour, communication and system factors (such as co-ordination of support service) as areas of concern, and time, lack of resources and nihilism as barriers to good care. [14,15] Several potential barriers to improved care were also identified as facilitators in our data. This suggests that these factors may be avoided or overcome. These included communication, workforce issues and system factors. Interestingly, successful improvements in the quality of care have not necessarily been accompanied by marked improvement in the knowledge of care providers, nor increases in their perception of the quality of care of care delivered. [16] Similarly, improved staff knowledge does not necessarily translate to improved care outcomes. [17] These findings are consistent with our data, which suggests that although knowledge *per se *may be important, problem solving, teamwork and communication skills will be critical components of successful interventions to improve the care of people with dementia. The data thus emphasise the need for future work to determine the best ways to support sustainable cultural change in residential care facilities and to allow facilities to identity and overcome local barriers to improved care. Organisational audit may be a useful tool for sites to identify local problems and assess their readiness for change.

Our respondents generally favored delivery of education in workshops, or workshops combined with other educational methodologies. These findings challenge the presumption that electronic methods of delivery will become increasingly popular. Generally, face-to-face workshops with a range of other educational methodologies have been the basis of effective educational interventions. [18] In contrast, education which relies simply on the provision of information may not be effective. [19] Further data are required to determine whether a preference for face-to-face delivery is because on-line methods have been found to be ineffective, or because they have not been trialed. Our findings, including preferences for multi-disciplinary training (shared between RCF staff and GPs), support the design of other proposed dementia training programs. [20]

To our knowledge this is the most comprehensive effort to understand perceived knowledge of dementia care in residential settings in Australia, and to identify the perceived factors relevant to improving care. In addition to RCF staff and GPs, family carers play a key role in the care of people with living in RCF. [21] Including the opinions of family carers increases the likelihood that the data are reliable and generalisable. [22] In addition to the multiple sources of data, the strong critical review process ensures that the data have face validity and increase the likelihood that the data are representative and externally valid. Consistency of themes between data from various sources (survey, focus groups and interviews) indicate that the data collected are internally valid and appear adequate in relation to the aims of the study.

Survey respondents were widely distributed geographically which increases the likelihood of representativeness of the sample. Results may be affected by volunteer bias. For example, newer staff (especially those who do not have strong English language skills) may be less likely to respond to surveys or volunteer to participate in interviews and focus groups. Staff perceiving their knowledge as good may be more likely to respond. The pattern of demographic characteristics of GPs attending RCF in our sample, compared to GPs who do not attend RCF, mirrored national data. Nationally, GPs attending RCF were more likely to be older and male (20% of male GPs attend RCF compared with 15% of female GPs; and 10% of GPs aged less than 35 years attend RCF compared with 12% of those aged 35–44 years, 20% aged 45–54 years and 22% aged 55 years or more). [6] This supports validity and representativeness of our survey sample. Despite this similarity in the pattern of demographic characteristics between our sample and the national data, the proportion of GPs attending RCF in our sample was higher than in the national data-set, suggesting a volunteer bias. GPs attending RCF may have been more likely to participate in our study. The proportion of GPs reporting participation in dementia education appears to be higher in our sample that in other overseas surveys which used more purposive sampling strategies. [23] Again this may reflect volunteer bias in our sample, rather than true differences.

Although the survey was comprehensively distributed, interpretation of the survey data is limited by the response rate. A limitation of the study was the absence of reminders or provision of a mechanism for follow up of surveys which were not returned. Preserving confidentiality was of high importance, making follow-up logistically challenging. The sampling strategy employed was considered the most feasible of the available options, but may have missed some potential participants (such as GPs who are not members of a Division). However these numbers are likely to be small. Our survey results should also be regarded with caution as the survey instrument has not been validated. We only measured perceived knowledge and did not validate respondents' perceived knowledge (such as by including knowledge based questions). Several other limitations warrant consideration. The survey was only distributed in English, and focus groups and interviews were only conducted in English. Whilst this would be less likely to be problematic for GPs and professional staff working in care facilities, it may have been a barrier to participation in the study for care staff. We did not distinguish accredited from unaccredited education sessions and this could be relevant to the quality of educational programs participated in, particularly for GPs, where a formal accreditation process is in place for continuing professional development activities.

## Conclusion

RCF staff and GPs attending RCF work together and frequently participate in dementia education programs, and current knowledge is perceived as good. However, many identify a gap in their knowledge and seek better understanding of how to provide quality care. Thus future educational interventions should be carefully designed to maximize engagement of all these groups. Interventions that facilitate learners assessing their prior knowledge and building on that foundation may be most likely to add value. Future educational interventions may need to be flexible to meet the needs of an increasingly heterogeneous and multicultural residential aged care workforce.

An educational strategy for RCF staff and GPs which is flexible, case-based, locally relevant and focused on practical strategies is most likely to meet the perceived needs of learners. Educational interventions need to consider sustainability, such as by identifying, nurturing and supporting local champions and leaders. Options for joint education of GPs and RCF staff should be provided. An educational program needs to include emphasis on the philosophy of care, and equipping participants with relevant skills, in addition to improving knowledge relating to dementia and dementia care. Further research is required to develop, implement and evaluate an educational intervention using these findings.

## Competing interests

The authors declare that they have no competing interests.

## Authors' contributions

CB, OPA, LF and NTL conceived the study. BH, SS, PF and FS contributed to study design. CB and BH carried out data handling and analysis in conjunction with study staff (see acknowledgements). CB drafted the manuscript. All authors contributed to data interpretation and critical review of the manuscript.

## Pre-publication history

The pre-publication history for this paper can be accessed here:


